# *Notes from the Field:* Multistate Outbreak of Eastern Equine Encephalitis Virus — United States, 2019

**DOI:** 10.15585/mmwr.mm6902a4

**Published:** 2020-01-17

**Authors:** Nicole P. Lindsey, Stacey W. Martin, J. Erin Staples, Marc Fischer

**Affiliations:** 1 Arboviral Diseases Branch, National Center for Emerging and Zoonotic Infectious Diseases, CDC, Fort Collins, Colorado.

Eastern equine encephalitis virus (EEEV), a mosquito-borne alphavirus, is the cause of one of the most severe arboviral diseases in North America (*1*). The clinical course typically begins as a systemic febrile illness but often progresses to neurologic disease (*2*). EEEV neuroinvasive disease is estimated to have a 30% case-fatality rate with approximately half of survivors left with neurologic sequelae (*2*,*3*). Although veterinary EEEV vaccines are available for use in horses, there are no licensed vaccines or effective treatments for humans. During 2003–2018, an average of eight EEEV disease cases were reported annually in the United States (range = 4–21 cases) (*3*,*4*). However, as of October 15, 2019, CDC received reports of 34 cases of EEEV disease from 21 counties in seven states (Figure). Cases were reported from Massachusetts (12 cases), Michigan (10), Connecticut (four), New Jersey (three), Rhode Island (three), North Carolina (one), and Tennessee (one). Dates of illness onset ranged from June 18 to September 20, 2019. Among the 34 patients, 21 (62%) had illness onset in August; 32 (94%) had a diagnosis of encephalitis, and two (6%) had a diagnosis of meningitis. Twenty-six (76%) patients were male. The median age was 64 years (range = 5–78 years); 21 (62%) of the 34 patients were aged ≥60 years.

**FIGURE:**
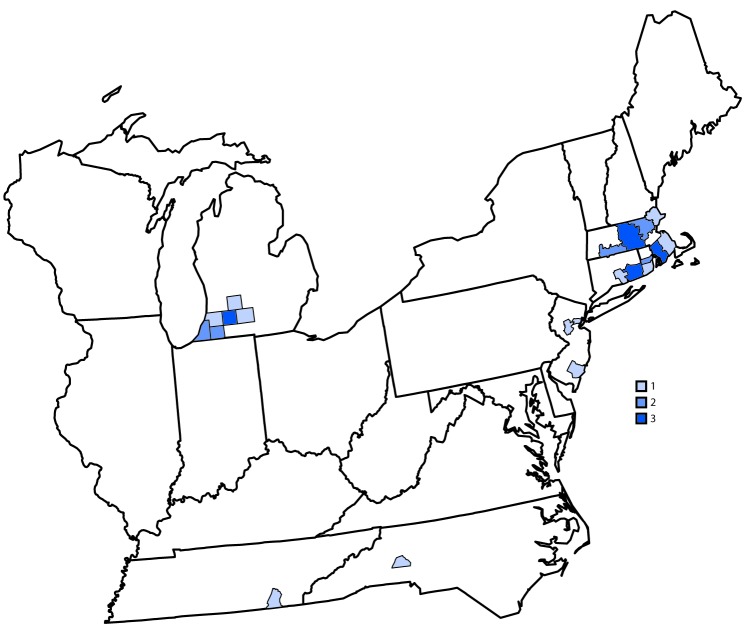
Number of reported cases of Eastern equine encephalitis virus disease (N = 34), by county of residence — United States, 2019* *As of October 15, 2019.

All 34 patients were hospitalized; 12 (35%) died. Deaths occurred a median of 12 days after illness onset (range = 4–38 days). Among the fatal cases, 10 (83%) patients were male, and the median age was 72 years (range = 58–78 years). The case-fatality ratio was highest among patients aged ≥70 years (seven of 11; 64%) and was 22% (five of 23) among patients aged <70 years.

EEEV is primarily maintained in an enzootic cycle between birds and *Culiseta melanura* mosquitoes, which breed in freshwater hardwood swamp environments in the eastern United States (*1*). Spread of EEEV to mammals typically requires mosquitoes (e.g., *Aedes* or *Coquillettidia* species) that feed on both birds and mammals (bridge vectors). Because of these complex interactions, the risk for human infection in a given year depends on multiple factors, including weather, abundance of birds and mosquitoes that can transmit the virus, human behavior, and clinical awareness and diagnostic testing practices (*5*).

It is not clear why more cases were reported in 2019 than in recent years. Larger outbreaks of EEEV occurred in several northeastern states in the 1930s and 1950s (*6**,**7*). These preliminary data for 2019 represent the largest number of cases reported in a single year since that time. However, changes in available diagnostic testing, populations at risk, national surveillance case definitions, and reporting systems make it difficult to compare annual case numbers before 2003.

Case counts in this report are provisional and might differ from those reported elsewhere. In areas at risk for EEEV transmission, health care providers should consider EEEV infection in the differential diagnosis of cases of aseptic meningitis and encephalitis and obtain appropriate serum or cerebrospinal fluid specimens for laboratory testing. Providers are encouraged to report suspected infections and send specimens to their state or local health department to facilitate diagnosis, increase public awareness, and potentially implement vector control to mitigate the risk for further transmission. Because human vaccines against EEEV are not available, prevention depends on community and household efforts to reduce vector populations (e.g., applying insecticides and reducing breeding sites) and personal protective measures to decrease exposure to mosquitoes (e.g., use of repellents and wearing protective clothing).
